# Knowledge, interest, and anticipated barriers of pre-exposure prophylaxis uptake and adherence among gay, bisexual, and men who have sex with men who are incarcerated

**DOI:** 10.1371/journal.pone.0205593

**Published:** 2018-12-07

**Authors:** Lauren Brinkley-Rubinstein, Meghan Peterson, Trisha Arnold, Amy S. Nunn, Curt G. Beckwith, Breana Castonguay, Eric Junious, Chantal Lewis, Philip A. Chan

**Affiliations:** 1 Department of Social Medicine, University of North Carolina, Chapel Hill, North Carolina, United States of America; 2 Center for Health Equity Research, University of North Carolina, Chapel Hill, North Carolina, United States of America; 3 Center for Prisoner Health and Human Rights, The Miriam Hospital, Providence, Rhode Island, United States of America; 4 Department of Psychology, Jackson State University, Jackson, Missouri, United States of America; 5 Rhode Island Hospital, Providence, Rhode Island, United States of America; 6 Department of Behavioral and Social Sciences, Brown University School of Public Health, Providence, Rhode Island, United States of America; 7 Department of Medicine, Brown University, Providence, Rhode Island, United States of America; 8 Center for AIDS Research, University of North Carolina, Chapel Hill, North Carolina, United States of America; 9 College of Health and Human Services, University of North Carolina, Charlotte, North Carolina, United States of America; Pontificia Universidade Catolica do Rio Grande do Sul, BRAZIL

## Abstract

Criminal justice (CJ) settings disproportionately include populations at high risk for acquiring HIV, and CJ-involved individuals are often at the intersection of multiple overlapping risk factors. However, few studies have examined attitudes about pre-exposure prophylaxis (PrEP) among incarcerated men who have sex with men (MSM). This study explored interest in, knowledge of, and barriers to PrEP uptake among gay, bisexual, and other men who have sex with men at the Rhode Island Department of Corrections. Using semi-structured interviews, 26 MSM were interviewed about PrEP knowledge, interest, timing preferences for provision (e.g. before or after release), and barriers to uptake and adherence during community re-entry. Interviews were coded and analyzed using a general inductive approach. Participants demonstrated low initial knowledge of PrEP but high interest after being told more about it. Participants self-identified risk factors for HIV acquisition, including condomless sex and substance use. In addition, participants preferred provision of PrEP prior to release. Post-release barriers to PrEP uptake and adherence included 1) concerns about costs of PrEP medications; 2) anticipated partner or family disapproval; 3) lack of access to transportation; 4) unstable housing; 5) compounding impacts of multiple hardships leading to a de-prioritization of PrEP and 6) fears of future re-incarceration. These results point to the need for future PrEP interventions among incarcerated populations that address incarceration and PrEP related barriers during community re-entry via wraparound services that address PrEP and incarceration-related barriers.

## Background

Criminal-justice (CJ) involved populations in the United States (US) are at increased risk for acquiring HIV. Rates of HIV among CJ-involved individuals are three times higher than in the general population [1] and one in seven HIV positive people pass through a local jail in any given year [1, 2]. Gay, bisexual, and other men who have sex with men (MSM) also have a heightened risk of HIV acquisition, which could be further amplified by substance use and other factors, including recent incarceration [3, 4]. The term “men who have sex with men” (MSM) refers to a heterogeneous group of men who have sex with members of the same sex, regardless of their sexual orientation. MSM who are CJ-involved are at increased risk for HIV infection, particularly Black MSM [5, 6]. In a study of 1,278 MSM in six US cities, incarceration rates were high, with 49% of individuals under the age of 30 reporting ever being incarcerated while 73% of those over 30 years reported ever being incarcerated [7]. For MSM, recent incarceration may disrupt relationships and contribute to sexual concurrency, resulting in higher HIV risk [8]. Some research has shown that HIV prevention programs implemented in CJ settings can be effective (e.g. condom distribution programs) [9, 10]. However, little research has focused on MSM who are currently incarcerated. Given that CJ-settings disproportionately include populations at high risk for acquiring HIV, they could be important venues to engage MSM in HIV prevention efforts.

A novel strategy for addressing HIV risk among CJ-involved MSM is exploring the potential use of pre-exposure prophylaxis (PrEP) while individuals are incarcerated or as they are preparing for release (with linkage to PrEP care in the community). PrEP is a bio-behavioral HIV prevention intervention that consists of an antiretroviral medication (emtricitabine/tenofovir, FTC/TDF) taken orally on a daily basis by HIV-uninfected individuals. PrEP’s efficacy has been well established in randomized controlled trials and open label studies [11–14]. The Centers for Disease Control and Prevention recommend PrEP be considered for at-risk populations including MSM [15]. In addition, the World Health Organization has introduced the concept of “substantive risk” as a precursor to PrEP indication. Those at substantive risk of HIV include any individual belonging to a group that has a disproportionate burden of HIV, which includes those with a history of incarceration and MSM [16].

A PrEP care continuum has been established that highlights the importance of PrEP awareness, access, linkage to PrEP care, uptake, adherence, and retention in care [17], and PrEP efficacy is closely tied to adherence [18]. Barriers to PrEP uptake are multi-level (individual, social, and structural) and include low awareness and knowledge of PrEP, perceived side effects, concerns about drug effectiveness, cost, access to PrEP prescriptions and medical care, perceived HIV-risk, stigma, and medical mistrust [19–27]. However, the degree to which individual, social, and structural barriers to PrEP uptake are exacerbated by incarceration and how they interact to impede use of PrEP among MSM with CJ experience is largely unknown. However, those with recent CJ-involvement often face substantial struggles during the post-release period such as loss of social support, inability to find housing or employment, and untreated mental illness or addiction [28, 29]. Our group has also found that medical and institutional distrust and lack of access to care may be barriers to engagement in HIV prevention programs [30]. Due in part to these kinds of barriers, PrEP uptake in real world settings such as CJ venues and after release remains low [31, 32].

In the current study, we interviewed 26 MSM who were incarcerated to explore PrEP knowledge, interest in taking PrEP, and potential barriers to uptake and adherence both in the correctional setting and the community. Given the lack of research that includes incarcerated MSM, this unique perspective underscores the significance of this study. In addition, to our knowledge, this study is among the first to investigate PrEP use, uptake, and adherence among incarcerated MSM.

## Methods

This study was conducted at the Rhode Island Department of Corrections (RIDOC) in Cranston, Rhode Island (RI). The RIDOC is a unified prison and jail through which approximately 15,000 men cycle each year [33]. The prevalence of HIV at the RIDOC is 3%, which mirrors correctional estimates of HIV prevalence in other jail or prison settings [34]. While RIDOC medical administration is interested in beginning a PrEP program, there is not yet one in place.

During medical intake, all individuals are asked about whether they self-identify as gay, straight, bisexual, or MSM by a RIDOC nurse. All individuals who reported being gay, bisexual, or MSM were approached and informed about the study. All possible participants that were approached had the ability to decline participation. Only one possible participant declined to participate. Due to the sensitive nature of this research, we requested a waiver of documentation of consent. This mean that participants only gave verbal consent prior to beginning the interview and the research team collected no identifiable information.

In addition, to being gay, bisexual, or MSM, study criteria included being over the age of 18, self-reporting as HIV negative, and ability to read and speak English. Semi-structured interviews lasted approximately 45–60 minutes and were conducted by trained qualitative researchers (LBR, CL, and MP). Interview topics included HIV knowledge, HIV risk perceptions, PrEP knowledge, PrEP interest, barriers/facilitators to taking PrEP while incarcerated, barriers/facilitators to taking PrEP in the community, and attitudes and suggestions about possible interventions. All interviews were conducted in a private room, digitally recorded and later transcribed. For participation, individuals received $30 that was deposited into their commissary account. Recruitment from the study occurred from September 2016 through October 2017. This study was approved by the Miriam Hospital’s institutional review board. The authors requested a waiver for documentation of consent. Therefore, all participants were informed about the study and only gave verbal consent to participate.

A general inductive approach guided the analysis, which allowed for the data to be formulated into themes and categories [35]. Coders (MP, LBR, and CL) read the transcribed data for participant responses that were similar and looked for recurrent themes and patterns in the transcripts. Open and axial coding were then used to outline concepts among two coders. Each theme and sub-theme was then assigned a code, and the codes were compiled in a codebook. Quality checks were conducted on 20% of all transcripts via iterative coding by at least two coders. At the beginning of the coding process coders separately coded transcripts and then went through each transcript to discuss discrepancies, consensus, and the creation of new, relevant codes. Discrepancies in interpretation were resolved among the research team before final coding commenced. Thematic saturation was achieved with the final sample.

## Results

Of the 26 participants, 16 were white, 8 were black, and 2 were Hispanic. Participants ranged in age from 23 to 57, and the average age was 38. None of the participants had ever been on PrEP before or tried to obtain it in the community. Across all interviews, key themes emerged related to 1) knowledge and interest in PrEP; 2) preferences for PrEP provision and 3) anticipated barriers to PrEP uptake and adherence during community re-entry.

### HIV risk and PrEP knowledge and interest

Many participants had never heard of PrEP, but some had limited knowledge about it. One participant stated: “I know that it’s a pill and people take it. I have, there’s some friends I know, acquaintances, that I’m the designated driver for all the time so I bring them places and they told me they were on PrEP and that it was easy, they felt safer now.”

Most participants also considered themselves at risk of acquiring HIV and discussed engaging in risky sexual behavior prior to incarceration. One participant stated: “You never really know who you’re sleeping with. Or what you’re sleeping with at the time or what that person has.” Another participant said: “There’s always the risk, like sometimes if I’m not having protected sex, if it’s just like one of those spur of the moment things it can happen any time, so. Well like not, not always, sometimes like I might not have a condom or you just think that everything will be fine and sometimes it’s not.”

In addition, several participants discussed addiction or drug use as an additional HIV risk factor or drug use as a contributor to sexual risk behavior. One participant stated: **“**I could also get in some bad stuff, sharing needles and stuff. Because I realized like I can relapse at any point, and then when I’m using drugs if it’s not intravenously I’m usually still more promiscuous, so like the risk of me getting HIV is just greater because I don’t care if I’m on drugs, I guess. And I’ve been relapsing so much lately.” Another participant also talked about his drug use in the community as a risk factor for engaging in risky sexual behavior: “I think I was for a while. I was very much in the scene where I mean I did crystal, I did a lot of party drugs and I was in a scene where sex was just everywhere.”

Correspondingly, most participants were also interested in taking PrEP. One participant stated:

“I’d be kind of proud doing something that would help me. It’s doing me well, even though I’m not doing the things its preventing me from doing. I’m helping myself if that time [to engage in risky behavior] does happen to come. It’s like putting the salt on your porch before it snows, so that the snow won’t ice up. It’s like preventing the slip and fall. Who wants to slip and fall? I’m helping myself from messing myself up.”

Another participant said:

“I know that at the end of the day if there’s anything that can prevent it or help prevent it, I say us as because whole like a family, you know what I mean, like the gay, transgender whatever like as a family we need to do whatever we can do to stop it, you know what I mean, before it gets too deep.”

Overall, participants had limited knowledge about PrEP prior to completing the interview, but the majority reported being at risk for HIV and acknowledged that their use of drugs contributed to their risk for HIV. Participants had a positive view of PrEP and most were interested in learning more or taking it.

### Preferences for PrEP provision

Participants were asked if they preferred to be prescribed PrEP prior to release from the RIDOC or if they would like to be linked to a PrEP clinic in the community. The majority of participants stated that they would like to receive a PrEP prescription and begin taking PrEP just prior to release from the RIDOC. Many also preferred to have a prescription and drug in hand (e.g. a 30-day PrEP pill pack) to take with them when they left. A participant stated: “[I’d like to get PrEP here] because I can get more information, first of all, I can be better with it, and I can be more effective if using in case I have any relationship no matter what here or outside, I’ll already be careful.”

However, even though most expressed that they would prefer to be prescribed PrEP at RIDOC, many stated that stigma and negative attitudes about, MSM, PrEP, and incarcerated individuals could be a barrier. A participant said:

“I guess the only thing that the only thing that would probably cause a problem is that if people knew that you were taking that medication and that that medication, it was strictly being targeted for guys that were having sex with guys there might be backlash between you know people that are homophobic.”

Another participant expressed a similar concern:

“Well, I think that the guards here don’t really take certain things seriously. You hear them cracking jokes about med line, about giving out meds that aren’t really needed. I’d worry on the administrative end that it wouldn’t be taken seriously enough as something that was actually needed.”

Participants reported that it would be easier to obtain a prescription for PrEP prior to release from incarceration. However, participants also voiced some concern about stigma associated with using PrEP while incarcerated and worried that PrEP use would lead to assumptions about their sexual orientation if other incarcerated individuals discovered they were taking it.

### Barriers to PrEP uptake and adherence during community re-entry

Given that PrEP is not currently available at the RIDOC or most other correctional facilities in the US, we asked participants what they thought might be barriers to PrEP uptake after release. Barriers mentioned by participants were PrEP-related, including cost and partner or family disapproval, and incarceration-related, including housing, transportation, the compounded effect of multiple hardships during community re-entry, and fear of reincarceration.

#### Barriers associated with PrEP use

Many participants expressed concern over taking PrEP. Most commonly cited were issues of cost and partner or family disapproval of PrEP use.

**Cost** While most participants were interested in taking PrEP, many wondered whether access to health insurance was necessary to assuage the high cost of the medication. For example:

“I mean just whether or not people’s health insurance would cover it, if that were to have an effect on it cause for me…if it were to be an expensive drug for people that if they had to pay for it without their health insurance helping them cover it then I think that that might be a hindering issue in getting it.”

**Disapproval of PrEP use** Several participants reported that their partners or family might not understand why they had decided to take PrEP. One participant stated:

“I have heard like about like ‘oh well that’s’ just for you know guys who want to hook up all the time who don’t want to have to worry about getting AIDS or HIV’, like that’s not really what it is, it’s a false, it’s a falsehood that you’re hearing, so I’d be afraid if like a family member of mine were to ask somebody about it and they asked somebody who wasn’t knowledgeable.”

Another person also ruminated on why someone might not feel comfortable if their partner knew they were taking PrEP:

**“**Let’s just say that you were a gay man with a partner and you are the type of person who cheats. You might not want your partner to know that you’re on it because you don’t want them to know that the reason you’re on it is because you’re having other sexual partners.”

#### Barriers associated with community re-entry

In addition to PrEP-related barriers, many of the barriers discussed by participants were related to incarceration. Specific issues mentioned by participants were lack of housing, transportation, and re-incarceration. In addition, several participants discussed how PrEP might necessarily be de-prioritized during community re-entry because compounding hardships that are exacerbated by incarceration must be addressed first.

#### Transportation

Many participants discussed how lack of transportation was a concern post-release that may affect PrEP uptake. When asked about barriers during community re-entry, one participant said: “And transportation [is an issue] because I lost my car getting to this craziness, like due to my charge. Like my car is under investigation, so that’s another thing that I’m really worried about.” Another participant stated: “A bus, a car, or some way to get there. Provided by anybody ‘cause I have a problem, my license run out so I can't drive.” Similarly, past research has identified lack of access to transportation as a barrier for recently incarcerated individuals to receiving HIV care.

#### Housing

Housing is often a concern for those leaving correctional institutions. Comments from participants mirrored these previous findings as most participants expressed worries about finding housing. For instance, a participant reported: “Well pretty much [I have] placement issues being that I’m not from Rhode Island.” Another stated: “I’m trying to find a place to live now. Cause I got nowhere.”

#### Compounded impact of multiple hardships during community re-entry and de-prioritization of PrEP

Many participants anticipated facing compounding hardships during community re-entry. Knowing that they would have many responsibilities and little resources after release, they wondered if PrEP would be a priority. A participant stated:

“When you go home you have so much to do. You have to go get your ID, and then you have to go back to welfare to get food stamps. You have so much different stuff you have to do for that first couple of days that you’re not really going to be thinking about I’ve got to go to the doctor’s [office] right away to get my PrEP. You have to go home and handle every other thing first.”

This same participant goes on to say:

“I mean every man goes through a couple of these, you know who’s going to be out there on the outside, who’s doing what, whether your friends are going to stick by you or not, whether they are going to leave you, and then there’s other worries like for those of us who have lost our apartments, where am I going to stay, you know all that stuff. That first day is kind of crucial to figure out things, and then if you don’t have anywhere to stay and you’re living with a friend temporarily you have that worry about getting your own place together, getting stuff together, security deposit, first month’s rent again. So, it’s a little bit of a mess.

Another participant discussed how PrEP may not be a priority during community re-entry: “Often times when a lot of us get out on the street we forget to do research, or we have so much going on that we don’t, or too much on our plates that it would probably be gone from my mind by the time I got off of it.”

Finally, another participant stated that while incarcerated people might prioritize PrEP, during post-release this may not be possible: “They [people who are incarcerated] have time to think you know and you want to participate in this and you want to do this, this, that, and you get that information, but people on the street don’t have time for that. They don’t want that. You know they, they want something that they give you and stuff like that. They don’t want to be part of that stuff.” In this quote, the participant is expressing the idea that while incarcerated individuals may have more time on their hands and would get involved in both research studies and engaging in other activities (like PrEP) but would not give these activities the same consideration in the community due to competing responsibilities.

Participants reported several competing hardships they were likely to experience post-release that may make getting information about PrEP and obtaining a prescription difficult. In addition, individuals acknowledged that if these hardships were not addressed they would likely be re-incarcerated: “If I don’t have another place to live I’m coming back. It’s just like you know the open-door policy is like a revolving door, you keep on going in and out, in and out, in and out.”

## Discussion

In this study, we evaluated knowledge, interest, and barriers to PrEP among CJ-involved MSM. Participant responses highlighted the ways in which PrEP can benefit those who are currently or recently CJ-involved but also illustrated the unique challenges to PrEP provision while incarcerated and uptake during community re-entry. The majority of participants expressed high interest in PrEP despite initial low knowledge, and many participants perceived themselves at high risk for acquiring HIV, citing overlapping sexual and substance use risk behaviors. While most participants expressed interest in beginning PrEP prior to release, they also emphasized how issues related to stigma might be a challenge to program implementation and PrEP uptake. Barriers to PrEP during community re-entry included lack of transportation, housing instability, cost, disapproval by peers and family, fears of re-incarceration and the de-prioritization of PrEP due to compounding hardships in the community.

Although no existing studies to our knowledge have focused on PrEP uptake among MSM with recent incarceration, our findings corroborate existing research related to both issues that are 1) challenges during community re-entry and 2) barriers to PrEP uptake and adherence in general. Participants reported housing instability and lack of transportation as significant barriers to PrEP uptake and adherence. The relationship between community re-entry and housing instability has been well documented [36–39]. Similarly, past research has identified lack of access to transportation as a barrier for recently incarcerated individuals to receiving HIV care [40–42]. Similarly, several PrEP uptake studies have identified cost and partner or family disapproval as barriers [43–46]. These issues are likely to present significant barriers to PrEP uptake and adherence among recently incarcerated MSM that may compound existing behavioral risk factors. Strategies to address and overcome these concerns are needed to implement effective PrEP programs in this population.

A unique finding of this study is the “de-prioritization” of PrEP post-release due to the overwhelming nature of multiple hardships that are present during community re-entry. Other studies have found that individuals may experience competing needs and that various multi-level factors may present barriers to engagement and retention in HIV care [47–50]. In our study, many participants pointed out that while they were interested in PrEP, they would have many practical issues to address that would come first. This highlights the need to consider establishing PrEP access prior to release either through referral (as a part of discharge services) to a PrEP clinic or, preferably, prescribing PrEP shortly before discharge. Prior to release individuals should have access to medical discharge planning services that ensure at-risk MSM can engage in comprehensive care that spans the PrEP continuum (uptake, adherence, and retention in care). Peer navigator programs that work with correctional staff to connect those at risk to PrEP programs in the community post-release may also be particularly useful. The use of peers has been a successful strategy for linking HIV positive individuals to care after release [51]. In addition, case management that links people to treatment for substance use and mental health may also aid in the alleviation of other community re-entry oriented burdens, and, thus, prevent incarceration-related barriers may prohibit PrEP uptake. In addition, PrEP education prior to release in the form of brief education classes, distribution of informational brochures, or one-on-one PrEP sessions with a medical professional may assuage concerns related to cost, as participants can learn about patient assistance programs, and develop strategies to deal with PrEP-related stigma.

Finally, it is worth underscoring that while the HIV risk inclusion criteria for this study was self-identifying as gay, bisexual or MSM, many participants found themselves at the intersection of risky drug use and sexual behavior. Therefore, future interventions targeting CJ-involved MSM should consider strategies that address sexual risk taking and substance use during community re-entry. This could include partnering PrEP interventions with medication assisted treatment programs that provide access to methadone, buprenorphine, or naltrexone in addition to HIV prevention services.

### Limitations

This study was conducted in a single state unified jail and prison system wherein a larger proportion of incarcerated individuals are White, which is very different from other states and may limit the generalization of our results. These findings are meant to only provide a formative understanding of PrEP among MSM in RI rather than be generalizable to all incarcerated MSM. However, future research should explore PrEP interest and attitudes among racial and ethnic minorities who are incarcerated. In addition, being included as a participant relied upon individuals’ self-report of being gay, bisexual or MSM, which may have many limitations. Individuals may have been unwilling to disclose that they were a MSM at the RIDOC due to anticipated stigma. Therefore, our sample is biased to only include those who felt comfortable disclosing. Finally, the current study does not explore the use of PrEP among other high risk incarcerated populations (e.g. those engaging in transactional sex or people who inject drugs).

## Conclusion

The current study is among the first to report on PrEP knowledge, interest, and barriers among CJ-involved MSM. Our results suggest that CJ-involved MSM are at the intersection of overlapping HIV risk factors, and that although knowledge of PrEP was low, significant interest exists. Barriers to PrEP include common community re-entry obstacles such as housing instability and lack of access to transportation and, general PrEP-related challenges such as cost and stigma. In addition, the multiple hardships often experienced during community re-entry compound and result in a de-prioritization of PrEP. Therefore, according to participant’s preference, future interventions should facilitate access to PrEP (either via referral or prescribing prior to discharge) before release and address barriers illuminated in this study.
